# Prolonged glucocorticoid use and rheumatoid arthritis-associated cervical spine deformity

**DOI:** 10.1007/s10067-025-07408-w

**Published:** 2025-04-04

**Authors:** Anna B. Lebouille-Veldman, Tom W. J. Huizinga, Rania A. Mekary, Carmen L. A. Vleggeert-Lankamp

**Affiliations:** 1 Department of Neurosurgery, Leiden University Medical Centre, Albinusdreef 2, 2300 RC Leiden, The Netherlands; 2 Department of Rheumatology, Leiden University Medical Centre, Leiden, The Netherlands; 3 Computational Neuroscience Outcomes Center at Harvard, Brigham and Women’s Hospital, Boston, MA USA; 4 MCPHS University, School of Pharmacy, 179 Longwood Avenue, Boston, MA 02115 USA; 5 Department of Neurosurgery, The Hague Medical Centre and HAGA Teaching Hospital, The Hague, The Netherlands; 6 Department of Neurosurgery, Spaarne Hospital Haarlem, Hoofddorp, The Netherlands

**Keywords:** Atlantoaxial subluxation, Cervical spine deformity, Glucocorticoid use, Rheumatoid arthritis, Vertical translocation

## Abstract

**Objectives:**

RA patients are often prescribed glucocorticoids, although it is known that their long-term use increases the risk of osteoporosis and fractures. The association between glucocorticoid use and RA-associated cervical spine deformity is yet to be determined.

**Method:**

Duration and dose of glucocorticoid use were evaluated in patients with new onset RA (BeSt Trial). Missing values on the exposure were imputed using the last observation carried forward. Lateral X-rays at 5- and 10-year follow-ups were assessed for atlantoaxial subluxation (AAS) and subaxial subluxation (SAS). To estimate the association between glucocorticoids and cervical spine deformity, multiple logistic regression models adjusted for age, gender, baseline Disease Activity Score (DAS), ACPA positivity, and RF positivity were used to estimate odds ratios (ORs) and their 95% confidence intervals (CIs). Mediation analysis was performed to evaluate whether such potential association was mediated via mean DAS.

**Results:**

Cervical deformity (AAS and/or SAS > 2 mm) was observed in 108 (40%) out of 272 patients. For a 1-year increase in total duration of glucocorticoid use, the adjusted OR for RA-associated cervical spine deformity was 1.19 (95% CI, 1.03–1.38; *p* = 0.02), and for an increase of 1 g of glucocorticoid in total cumulative dose, the OR was 1.06 (95% CI, 1.01–1.12; *p* = 0.02). Mediation analysis could not reveal an influence of mean DAS on these associations.

**Conclusions:**

There was evidence of a direct association between long-term use of glucocorticoids in newly diagnosed RA patients and RA-associated cervical spine deformity after 10 years. Other effective therapies to suppress disease activity may be preferred over glucocorticoids.

**Table:** 

**Key Points** • *For a 1-year increase in total duration of prednisone use in RA patients, the adjusted OR for RA-associated cervical spine deformity was 1.17 (95% CI, 1.01–1.36; p = 0.04).* • *For an increase in total cumulative dose of 1 g of prednisone in RA patients, the adjusted OR for RA-associated cervical spine deformity was 1.06 (95% CI, 1.00–1.11; p = 0.04).* • *The use of glucocorticoids in RA patients was associated with an increased odds of RA-associated cervical spine deformity after 10 years, which may suggest that other effective therapies to suppress disease activity should be preferred over glucocorticoids.*

## Introduction

Glucocorticoids play a significant role in the treatment of rheumatoid arthritis (RA). This inflammatory disease, which occurs in about 5 per 1000 people, is known for its debilitating effect on the daily life of patients [1]. This leads to a decreased health-related quality of life and an increased mortality [2]. For decades, glucocorticoids have been used in the treatment of RA. The inflammation-reducing effect of this medication is used to prevent or at least diminish damage progression to the joints [3]. Glucocorticoids are often prescribed to initiate decrease of systemic inflammation in case of an RA inflammatory episode. Many patients use low doses of glucocorticoids for prolonged periods of time to suppress systemic disease activity. It has been shown that long-term use of low doses of glucocorticoids substantially reduces the progression of erosions in the hands and feet of RA patients [4, 5].

It has been a clinical observation in the daily management of patients with RA, that in the last decades severe damage to the cervical spine occurs far less frequently than before. It has been hypothesized that damage to the spine, as damage to peripheral joints, occurs less often because rheumatic disease activity can nowadays be better suppressed, using (newer and combinations of) antirheumatic drugs, earlier in the disease course and following principles of tight control and treatment-to-target. Glucocorticoids as treatment for RA may help to suppress disease activity. However, the use of glucocorticoids, in particular in higher doses over longer periods of time, is associated with risks for adverse effects, such as osteoporosis, infections, and cardiovascular disease [6–9]. It should be considered though that these adverse effects are also common in the natural course of rheumatoid arthritis [10–12]. Therefore, the 2021 American College of Rheumatology guidelines recommend to start treatment of RA without use of glucocorticoids, although the voting panel members acknowledge that short-term glucocorticoids are frequently necessary [2, 13]. The EULAR 2022 recommendations for the management of RA state that glucocorticoids should be considered when initiating or changing DMARDs, but that they should be tapered and stopped as rapidly as clinically feasible, preferably within 3 months after initiation.

Besides the recommendation against glucocorticoid use based on toxicity, knowledge on the effect of (prolonged) glucocorticoid use on the cervical spine is scarce. Cervical spine deformities in RA can consist of atlantoaxial subluxation (AAS), subaxial subluxation (SAS), and/or vertical translocation (VT). These deformities can cause severe symptoms such as pain in the neck and the occipital region, as well as neurological symptoms due to medullary compression, potentially even leading to paralysis or death [14]. In older RA cohorts, glucocorticoid use has been shown to be associated with presence of cervical spine deformities [15], but the association with DAS was not systematically established [16]. Moreover, in the BeSt Trial, no association between cervical deformity and mean DAS, DAS over time [17], or flares of DAS was observed [18]. As with other outcomes, the risks for adverse effects of glucocorticoids may be balanced with benefits of suppression of RA inflammation with glucocorticoids. Therefore, we set out to investigate the relationship between each of dose and duration of use of glucocorticoids and cervical deformity after a period of 10-year follow-up in patients with newly diagnosed RA.

## Methods

### Study design

This retrospective case–control study used data from the BeSt trial, which is a single-blinded multicenter randomized clinical trial (RCT), designed to compare four treatment strategies in patients with early active RA (all then fulfilling the American College of Rheumatology 1987 classification criteria), with at least 6 inflamed joints (of 66 assessed) and either a high erythrocyte sedimentation rate or a high patient rating of disease activity [19]. Patients were recruited in 18 non-university and two university hospitals in The Netherlands between 2000 and 2002. There were 508 patients enrolled in the original study. The Medical Ethics Committee of the LUMC approved the study (P08.011) and the regulatory boards of the individual hospitals approved likewise. All patients gave written informed consent.

In the original RCT, patients were randomized to the following treatment arms: (1) sequential monotherapy (starting with methotrexate monotherapy); (2) step-up combination therapy (also starting with methotrexate monotherapy); (3) initial combination therapy with methotrexate, sulfasalazine and prednisone; and (4) initial combination therapy with methotrexate and infliximab. All patients were treated according to the “treat-to-target” principle, requiring protocolized treatment adjustments based on three-monthly assessments of the Disease Activity Score (DAS, based on a 44 (for swelling)/53 (for tenderness using the Ritchie Articular Index) joint count, ESR and patient’s assessment of disease activity). In case of a DAS > 2.4, treatment was increased according to the next step in the relevant treatment strategy arm. Subsequent steps for patients with an insufficient response included prednisone in all four treatment regimens. If the treatment resulted in a lower disease activity, the strategy aimed to taper medication and to finally achieve drug-free remission [19]. 

### Ascertainment of cases (patients with RA-associated cervical spine deformity) and controls (patients without RA-associated cervical spine deformity)

Lateral X-rays of the cervical spine were collected at 5- and 10-year follow-up. No baseline cervical spine radiographs were available. Radiological cervical deformity parameters (AAS, SAS, and VT) were evaluated on lateral X-rays by two researchers (ABV and CVL), both blinded for patient characterstics [17]. Agreement was reached in close cooperation. If a dynamic X-ray was performed (flexion/extension), AAS was scored separately on these radiographs. In the main analysis, cases of cervical spine deformity of all types (mild, moderate, or severe) were defined as the presence of AAS and/or SAS > 2 mm. AAS was defined as a distance of more than 2 mm between the odontoid peg and the anterior arch of C1 in neutral position. SAS was concluded to be present in case a listhesis of more 2 mm existed in neutral position. In sensitivity analyses, AAS ≥ 3 mm in flexion on dynamic X-ray was a second outcome in this study, which included moderate and severe cervical spine deformity. AAS ≥ 5 mm in flexion or neutral position was a third outcome, which included only severe cervical spine deformity. VT was present if the tip of the odontoid peg exceeded the line of McGregor [20]. If an X-ray was missing at 10-year follow-up and AAS, VT or SAS was present at 5-year follow-up; it was scored to be also present at 10-year follow-up. For the AAS ≥ 3 mm in flexion and severe AAS groups, if a flexion X-ray was missing at 10-year follow-up and AAS was not present at 5 years, the patient was not included in the sub analysis for these groups.

In controls, no RA-associated cervical spine deformity was present and patients were derived from the same population that gave rise to the cases.

### Exposure assessment: use and dose of glucocorticoids in the trial

Patients received prednisone or methylprednisolone either per protocol, as part of the step-up treatment strategy, or as a protocol violation, in the form of intra-articular injection, if deemed necessary by the treating physician in addition to the randomized treatment. The use and dose of glucocorticoids (prednisone and methylprednisolone) were assessed every 3-month visits during the 10-year evaluation period. Doses were calculated as a total dose of prednisone, using the Steroid Conversion Calculator from MDCalc [21]. Because of the long follow-up, some patients were followed on a yearly basis, when possible, because they were no longer willing to be seen quarterly. The amount of missing data on glucocorticoid use was 9.2% (25 patients) during any timepoint. In this case, imputation in the form of Last Observation Carried Forward (LOCF) was used. To investigate the association between glucocorticoids and cervical spine deformity at 10-year follow-up, both duration and cumulative dose of glucocorticoids used during 10 years were studied separately. The cumulative dose of prednisone during 10 years was calculated as the total number of grams used during 10 years follow-up.

### Measurement of covariates

Age, sex, ACPA status, and rheumatoid factor status of the patients were collected at baseline. Treatment strategy was determined after randomization between four treatment strategies [19]. During a period of 10 years, the disease activity score (DAS) and Health Assessment Questionnaire (HAQ) scores were measured every 3 months and thus 41 times in total. In case of missingness of DAS values, multiple imputation was used. The imputation model included terms for treatment strategy, age, sex, ACPA status, rheumatoid factor status, HAQ, and DAS values. In the imputation, 20 iterations were pooled using MATLAB 2019b and combined to form the individual DAS data that were used to explore correlations, and also to form a mean DAS value [17].

### Exclusion of patients at baseline

Of the 311 patients included in the original RCT who underwent imaging of their cervical spine, 39 patients were excluded because they were missing the X-ray at 10 years and had no signs of cervical deformity at 5-year follow-up. Ultimately, in this study, 272 patients had adequate radiological (radiographs at 10-year follow-up present or cervical deformity present in the 5-year follow-up neutral radiographs) and DAS44 follow-up data, and were hence included in the case–control study [17].

### Statistical analysis

Baseline demographic data were expressed as mean ± SD, as well as the total dose of glucocorticoids administered during the 10-year follow-up period. Multiple logistic regression was performed to study the association between each of increased duration of glucocorticoid use in years as well as increased cumulative dose of glucocorticoids in relation to different outcomes of cervical spine deformity. These models were adjusted for potential confounding variables such as age, gender, DAS at baseline, ACPA status, and RF status.

The treatment protocols dictated the addition of glucocorticoids in patients with higher systemic disease and thus higher DAS values. Because of this, more glucocorticoids were prescribed in patients with higher DAS values. To be able to discern the effect of glucocorticoids from the effect of DAS on cervical spine deformity, a mediation analysis was performed, using the mean DAS value over the 10-year follow-up as mediator. Notably, previous analyses have demonstrated that an association between DAS and cervical spine deformity was absent [17], but we still deemed it useful to attempt to study the influence of systemic disease activity on our results. First, using linear regression for continuous outcomes and binomial logistic regression for binary outcomes, the associations between increased duration of glucocorticoid use and cervical spine deformity (binary); between glucocorticoid use and the mediator (mean DAS value, continuous); and between the mediator and cervical deformity (binary) were assessed. Then, the potential effect of the mediator was determined by assessing the magnitude of any potential change in the OR and 95% CI between the regression analyses without and with the mediator as an added factor. Statistical analyses were performed using SPSS version 29.1 and MATLAB version 2019b [22, 23]. A two-tailed *p*-value < 0.05 was deemed statistically significant.

## Results

After 10 years, 62 of 272 patients, (23%) had Atlantoaxial subluxation (AAS) of more than 2 mm in neutral position. Of the 272 patients, 60 (22%) had subaxial subluxation (SAS). No patients had VT above the line of McGregor. In total, 108 of 272 patients (40%) had RA-associated cervical spine deformity of any kind on neutral X-ray. In this latter group of patients, the mean age at baseline was 55.2 ± 12.7 years, 60% were females, 69% were RF positive, and 68% were ACPA positive. This was comparable to the demographic data of the patients without cervical spine deformity, except for age; at 10-year follow-up, patients with cervical spine deformity appeared older than patients without cervical spine deformity (Table 1).

**Table 1:** Baseline characteristics of rheumatoid arthritis patients with or without cervical spine deformity at 10-year follow-up

Baseline characteristics	Cervical spine deformity at 10-year follow-up (*n* = 108)	No cervical spine deformity at 10-year follow-up (*n* = 164)
Mean age (± SD)	55.2 (± 12.7)	50.6 (± 11.2)
Female, *n* (%)	65 (60%)	117 (71%)
Mean DAS (± SD)	4.39 (± 0.89)	4.34 (± 0.86)
Mean HAQ score (± SD)	1.22 (± 0.66)	1.36 (± 0.63)
RF positive, *n* (%)	74 (69%)	112 (68%)
ACPA positive, *n* (%)	73 (68%)	104 (63%)

^*^Abbreviations: *DAS*, disease activity score; *HAQ*, health assessment questionnaire; *RF*, rheumatoid factor; *ACPA*, anti-citrullinated protein antibodies

An X-ray in flexion was available for 109 patients. Of these, 26 patients (24%) demonstrated AAS ≥ 3 mm in flexion. Two of these patients demonstrated AAS ≥ 5 mm in flexion. Additionally, on the neutral X-rays, 6 patients demonstrated an AAS ≥ 5 mm. Consequently, a total of 8 patients (3%) had AAS ≥ 5 mm in flexion and/or neutral position (severe AAS).

### Glucocorticoid use

Of the 272 patients with 10-year cervical spine radiographs, 147 (54%) used glucocorticoids for some duration during the 10-year follow-up. The daily glucocorticoid dose ranged between 1.1 and 30 mg. Among the 108 cases of patients with cervical spine deformity, a mean cumulative dose of 4.21 ± 6.85 g with 57 patients (53%) who used glucocorticoids at any time during follow-up and a median duration of glucocorticoid use of 4.5 months (IQR 0–21). However, among the 164 controls, these numbers were slightly lower with a mean cumulative dose of 2.59 ± 3.99 g, 90 patients (55%) who used glucocorticoids at any time during follow-up, and a median duration of glucocorticoid use of three months (IQR 0–15).

### Association between glucocorticoid duration and RA-associated cervical spine deformity

After adjustment for potential confounders including age, gender, DAS at baseline, ACPA status, and RF status, a 1-year increase in duration of glucocorticoid was associated with a statistically significant 19% increased odds for the presence of all cervical spine deformity (AAS and/or SAS > 2 mm) (OR of 1.19; 95% CI, 1.03–1.38; *p* = 0.02), a non-statistically significant odds ratio of 1.24 (OR, 1.24; 95% CI, 0.92–1.67; *p* = 0.16) for the presence of moderate or severe cervical spine deformity (AAS ≥ 3 mm in flexion), and a statistically significant 57% increased odds (OR, 1.57; 95% CI, 1.09–2.28; *p* = 0.02) for the presence of severe cervical spine deformity (AAS ≥ 5 mm in flexion or neutral). Adding average DAS during the 10-year follow-up did not materially change the odds ratios and their 95% confidence intervals for each of the three outcomes (Table 2).

**Table 2:** Multiple logistic regression for duration of glucocorticoid use in relation to mild, moderate, or severe cervical spine deformity at 10-year follow-up

	Multivariable odds ratio (95% confidence interval)	*P*-value
Model 1 for cases of mild, moderate, or severe (AAS > 2 mm and/or SAS > 2 mm) (n_1_ = 272)		
Duration of glucocorticoid use (years)†	1.19 (1.03, 1.38)	0.02
Duration of glucocorticoid use (years) + average DAS score‡	1.17 (1.01, 1.36)	0.04
Model 2 for cases of moderate or severe (AAS ≥ 3 mm in flexion) (n_2_ = 109)		
Duration of glucocorticoid use (years) †	1.24 (0.92, 1.67)	0.16
Duration of glucocorticoid use (years) + average DAS score‡	1.26 (0.92, 1.72)	0.16
Model 3 for severe (AAS ≥ 5 mm in flexion or neutral) (n_3_ = 107)		
Duration of glucocorticoid use (years) †	1.57 (1.09, 2.28)	0.02
Duration of glucocorticoid use (years) + average DAS score‡	1.53 (1.05–2.24)	0.03

^**†**^All three models for the three different outcomes of cervical spine deformity (AAS or SAS > 2 mm; AAS ≥ 3 mm; AAS ≥ 5 mm) were adjusted for age at baseline, sex (male or female), anti-CCP positivity, rheumatoid factor positivity, and baseline DAS score

^‡^Adjusted for the previous multivariable model plus mean DAS during the 10-year follow-up

^*^Abbreviations: *DAS*, Disease Activity Score; *AAS*, Atlantoaxial Subluxation; *SAS*, subaxial subluxation

### Association between cumulative dose and cervical spine deformity

After adjustment for potential confounders including age, gender, DAS at baseline, ACPA status, and RF status, there was a strong evidence that a one-gram increase in cumulative dose of glucocorticoids was associated with a statistically significant 6% increased odds for the presence of all cervical spine deformity (AAS and/or SAS > 2 mm) (OR of 1.06; 95% CI, 1.01–1.12; *p* = 0.02), a non-statistically significant odds ratio of 1.05 (OR of 1.05; 95% CI, 0.93–1.18; *p* = 0.45) for the presence of moderate or severe cervical spine deformity (AAS ≥ 3 mm in flexion), and a statistically significant 22% increased odds (OR of 1.22; 95% CI, 1.05–1.43; *p* = 0.01) for the presence of severe cervical spine deformity (AAS ≥ 5 mm in flexion or neutral). Adding average DAS during the 10-year follow-up did not materially change the odds ratios and their 95% confidence intervals for each of the three outcomes (Table 3).

**Table 3:** Multiple logistic regression for cumulative dose of glucocorticoids used in relation to mild, moderate, or severe cervical spine deformity at 10 years follow-up

Variable and sample size	Multivariable odds ratio (95% confidence interval)	*P*-value
Model 1 for cases of mild, moderate, or severe (AAS > 2 mm and/or SAS > 2 mm) (n_1_ = 272)		
Cumulative dose of glucocorticoids (grams)†	1.06 (1.01, 1.12)	0.02
Cumulative dose of glucocorticoids (grams) + average DAS score‡	1.06 (1.00, 1.11)	0.04
Model 2 for cases of moderate or severe (AAS ≥ 3 mm in flexion) (n_2_ = 109)		
Cumulative dose of glucocorticoids (grams)†	1.05 (0.93, 1.18)	0.46
Cumulative dose of glucocorticoids (grams) + average DAS score‡	1.05 (0.92, 1.19)	0.48
Model 3 for severe (AAS ≥ 5 mm in flexion or neutral) (n_3_ = 107)		
Cumulative dose of glucocorticoids (grams)†	1.22 (1.05, 1.43)	0.01
Cumulative dose of glucocorticoids (grams) + average DAS score‡	1.21 (1.03, 1.42)	0.02

^**†**^All three models for the three different outcomes of cervical spine deformity (AAS or SAS > 2 mm; AAS ≥ 3 mm; AAS ≥ 5 mm) were adjusted for age at baseline, sex (male or female), anti-CCP positivity, rheumatoid factor positivity, and baseline DAS score

^‡^Adjusted for the previous multivariable model plus mean DAS during the 10-year follow-up

^*^Abbreviations: *DAS*, Disease Activity Score; *AAS*, atlantoaxial subluxation; *SAS*, subaxial subluxation

## Discussion

A direct association was observed for both duration and total cumulative dose of glucocorticoids with RA-associated cervical spine deformity, when adjusted for possibly confounding factors such as age, gender, baseline DAS, ACPA status, and RF status. Ever use of glucocorticoid was similar in patients with and without cervical spine deformities, but the cumulative duration and cumulative dose of glucocorticoid use were higher in patients with cervical spine deformities.

These results suggest that while glucocorticoids may be effective in suppressing disease activity, prolonged use may have a negative effect on ligaments around the upper cervical spine structures to contribute to development of cervical spine deformities. This is surprising as at other sites prone to damage by rheumatoid inflammation, no negative effects of glucocorticoids have been reported. Other publications concerning the described treatment strategies revealed that better suppression of disease activity is associated with less joint damage in hands and feet [10–12]. We looked for other studies to shed more light on this matter; however, limited literature is available on the association between prolonged glucocorticoid use and RA-associated cervical spine deformity, and especially from patients with recent onset RA that were treated to target. A 2017 meta-analysis by Zhu et al. on 12 studies discussing risk factors associated with cervical spine deformity in RA has shown a pooled OR of cervical spine involvement in 2750 RA patients of 2.2 (95% CI, 1.7–2.8, *p* < 0.001) with increased glucocorticoid use [15]. However, no specification was given on the dose and/or duration of glucocorticoids use. The study defined AAS as present if the atlantodental interval exceeded 3 mm, VT as present if the Ranawat value < 13 mm or Redlund-Johnell value < 29 mm, and SAS as present if subluxation was present ≥ 2 mm. These definitions are best compared to our definitions of AAS ≥ 3 mm in flexion and severe deformity, and conclusions do fit well with ours.

A Japanese prospective multicenter cohort study from 2001 with a follow-up time up to 12 years that clinically evaluated patients with established RA and assessed cervical spine radiographs at cohort entry and final visit found that poor functional ability, rapid functional deterioration, and glucocorticoid use were associated with development or worsening of cervical deformations [24]. In a Korean study comparing RA patients with neck pain with and without cervical deformities, higher use of glucocorticoids (unknown dose), but also use of methotrexate, combinations of DMARDs, longer symptom duration, high ESR, and presence of erosions at radiographs of hands and feet were observed in patients with cervical spine involvement (AAS > 2.5 mm, SAS > 1 mm and VT if the tip of the dens translated > 4.5 mm above McGregor) [25]. An older study including 134 RA patients, being evaluated for a period up to 10 years, showed more glucocorticoid use in the 92 patients with cervical deformations than in the 42 patients without cervical deformations. No details about disease activity were provided [26].

The majority of cervical spine deformities demonstrated in this study were mild. Therefore, the current place that glucocorticoids should have in the treatment of RA has not unequivocally been clarified. It can be concluded though that long duration of use and/or high dosage may do more harm than good concerning cervical deformity. In practice, glucocorticoids are used to treat patients with high disease activity and they can be very effective for that purpose. However, if glucocortoid use can be prevented, especially long-term and in high doses, it could lower the odds of cervical spine deformity in the long term.

A limitation of our analyses is the difficulty in differentiation between the effect of disease activity on damage and the effect of prednisone on disease activity and potentially on damage. Even though we adjusted for baseline disease activity as potential confounder in regression analyses, this may not be sufficient. For instance, DAS at baseline did not influence the initial high tapered prednisone dose scheme in arm 3, which was dependent on randomization. Also, during treatment, prednisone dose changes were not only dependent on current DAS but also by the previous prednisone dose as dictated by the treatment protocol. The treatment protocol itself set limitations over time to repeat the use of prednisone in the course of the study. Moreover, DAS over time may not sufficiently capture the specific effect of episodes of high DAS or very low DAS (remission), which by themselves have affected subsequent prednisone use or a switch to other anti-inflammatory treatments. Nevertheless, the mediation analysis could not demonstrate an influence of mean DAS on the cervical deformity at the end of follow-up.

Another limitation was the lack of X-rays in flexion, extension, and neutral position of all patients at baseline and for some patients at 5- and 10-year follow-up. X-rays were occasionally challenging to interpret and quantify. The X-rays were made in the workflow of a study aiming at clinical parameters and the evaluations were not done instantly. In daily practice, if an X-ray of the cervical spine in RA patients is performed, it is evaluated carefully and, if difficult to interpret, made again. That would have yielded more qualitative X-rays in some cases, and a better follow-up over the years. Furthermore, particularly excluding the patients missing the 10-year follow-up X-ray after their 5-year X-ray demonstrated no deformity might have induced selection bias. This could lead to an underestimation of the true presence of deformity, as these patients may have developed cervical deformity in the 5- to 10-year follow-up period. Additionally, because of the long follow-up period, there were some missing data in this study. We used imputation in the form of the last observation carried forward for missing data points in glucocorticoid use. While we think this method best fits our data and the type of data imputed, this may introduce some bias.

Despite these limitations, our study had several strengths. This study had a long follow-up duration of 10 years, where patients with early-onset RA were followed and assessed at quarterly intervals. In addition, we adjusted for multiple possible confounding variables, used multiple imputations for missing values in average DAS, and performed a mediation analysis for DAS during the 10-year follow-up.

In conclusion, patients with cervical spine deformities had a longer duration and a higher dose of glucocorticoid than patients without cervical deformities. There was evidence of a direct association between each of a year increase of use and a one-gram increment of cumulative dose of glucocorticoids and cervical deformity after 10 years of follow-up. So, while glucocorticoids are known to have a beneficial effect on overall disease activity, caution should be used in prescribing glucocorticoids for long time periods in patients at risk for developing cervical spine deformity. Future studies should focus on finding factors to predict the development of cervical spine deformity in RA patients. Particularly in those patients, other effective treatments, such as biological DMARDs, should be initiated instead when possible and appropriate [13].

## References

[1] Aletaha D, Smolen JS (2018) Diagnosis and management of rheumatoid arthritis: a review. JAMA 320(13):1360–1372. 10.1001/jama.2018.13103 30285183 10.1001/jama.2018.13103

[2] Singh JA, Saag KG, Bridges SL Jr et al (2016) 2015 american college of rheumatology guideline for the treatment of rheumatoid arthritis. Arthritis Rheumatol 68(1):1–26. 10.1002/art.39480 26545940 10.1002/art.39480

[3] van der Goes MC, Jacobs JW, Bijlsma JW (2014) The value of glucocorticoid co-therapy in different rheumatic diseases–positive and adverse effects. Arthritis Res Ther 16(Suppl 2). 10.1186/ar4686. (S2) 10.1186/ar4686 PMC4249491 25608693

[4] Kirwan JR, Bijlsma JW, Boers M, Shea BJ (2007) Effects of glucocorticoids on radiological progression in rheumatoid arthritis. Cochrane Database Syst Rev 2007(1):CD006356. 10.1002/14651858.CD006356 17253590 10.1002/14651858.CD006356 PMC6465045

[5] Boers M, Hartman L, Opris-Belinski D et al (2022) Low dose, add-on prednisolone in patients with rheumatoid arthritis aged 65+: the pragmatic randomised, double-blind placebo-controlled GLORIA trial. Ann Rheum Dis 81(7):925–936. 10.1136/annrheumdis-2021-221957 35641125 10.1136/annrheumdis-2021-221957 PMC9209692

[6] del Rincon I, Battafarano DF, Restrepo JF, Erikson JM, Escalante A (2014) Glucocorticoid dose thresholds associated with all-cause and cardiovascular mortality in rheumatoid arthritis. Arthritis Rheumatol 66(2):264–272. 10.1002/art.38210 24504798 10.1002/art.38210

[7] Bijlsma JWJ, Buttgereit F (2016) Adverse events of glucocorticoids during treatment of rheumatoid arthritis: lessons from cohort and registry studies. Rheumatology (Oxford) 55(suppl 2):ii3–ii5. 10.1093/rheumatology/kew344 27856654 10.1093/rheumatology/kew344

[8] Curtis JR, Westfall AO, Allison J et al (2006) Population-based assessment of adverse events associated with long-term glucocorticoid use. Arthritis Rheum 55(3):420–426. 10.1002/art.21984 16739208 10.1002/art.21984

[9] Huscher D, Thiele K, Gromnica-Ihle E et al (2009) Dose-related patterns of glucocorticoid-induced side effects. Ann Rheum Dis 68(7):1119–1124. 10.1136/ard.2008.092163 18684744 10.1136/ard.2008.092163

[10] Palmowski A, Nielsen SM, Boyadzhieva Z et al (2023) Safety and efficacy associated with long-term low-dose glucocorticoids in rheumatoid arthritis: a systematic review and meta-analysis. Rheumatology (Oxford) 62(8):2652–2660. 10.1093/rheumatology/kead088 36810945 10.1093/rheumatology/kead088

[11] Wiebe E, Huscher D, Schaumburg D et al (2022) Optimising both disease control and glucocorticoid dosing is essential for bone protection in patients with rheumatic disease. Ann Rheum Dis 81(9):1313–1322. 10.1136/annrheumdis-2022-222339 35680387 10.1136/annrheumdis-2022-222339 PMC9380479

[12] Bergstra SA, Sepriano A, Kerschbaumer A et al (2023) Efficacy, duration of use and safety of glucocorticoids: a systematic literature review informing the 2022 update of the EULAR recommendations for the management of rheumatoid arthritis. Ann Rheum Dis 82(1):81–94. 10.1136/ard-2022-223358 36410794 10.1136/ard-2022-223358

[13] Fraenkel L, Bathon JM, England BR et al (2021) 2021 American college of rheumatology guideline for the treatment of rheumatoid arthritis. Arthritis Care Res (Hoboken) 73(7):924–939. 10.1002/acr.24596 34101387 10.1002/acr.24596 PMC9273041

[14] Kauppi MJ, Barcelos A, da Silva JA (2005) Cervical complications of rheumatoid arthritis. Ann Rheum Dis 64(3):355–358. 10.1136/ard.2003.020230 15708890 10.1136/ard.2003.020230 PMC1755389

[15] Zhu S, Xu W, Luo Y, Zhao Y, Liu Y (2017) Cervical spine involvement risk factors in rheumatoid arthritis: a meta-analysis. Int J Rheum Dis 20(5):541–549. 10.1111/1756-185X.13096 28524646 10.1111/1756-185X.13096

[16] Veldman AB, Allaart CF, Vleggeert-Lankamp CLA (2022) The influence of reducing disease activity score on cervical spine deformity in rheumatoid arthritis: a systematic review. Biomed Res Int 2022:9403883. 10.1155/2022/9403883 35463987 10.1155/2022/9403883 PMC9033349

[17] Lebouille-Veldman AB, Spenkelink D, Allaart CF, Vleggeert-Lankamp CLA (2023) The association between disease activity score and rheumatoid arthritis-associated cervical deformity: radiological evaluation of the BeSt trial. J Neurosurg Spine 1–8. 10.3171/2022.12.SPINE221057 10.3171/2022.12.SPINE221057 36640102

[18] Lebouille-Veldman AB, Allaart CF, Vleggeert-Lankamp CLA (2025) Cervical spine deformity and flares in disease activity in rheumatoid arthritis. Brain and Spine 5:104235. 10.1016/j.bas.2025.104235 10.1016/j.bas.2025.104235 PMC11979421 40206593

[19] Markusse IM, Akdemir G, Dirven L et al (2016) Long-term outcomes of patients with recent-onset rheumatoid arthritis after 10 years of tight controlled treatment: a randomized trial. Ann Intern Med 164(8):523–531. 10.7326/m15-0919 27089068 10.7326/M15-0919

[20] Kim DH, Hilibrand AS (2005) Rheumatoid arthritis in the cervical spine. J Am Acad Orthop Surg 13(7):463–474. 10.5435/00124635-200511000-00006 16272271 10.5435/00124635-200511000-00006

[21] MDCalc. https://www.mdcalc.com/calc/2040/steroid-conversion-calculator#why-use. Accessed 24 July 2024

[22] IBM Corp. Released 2021. IBM SPSS Statistics for Windows, Version 28.0. Armonk, NY: IBM Corp

[23] Inc. TM. MATLAB version 2019b. The MathWorks Inc. https://www.mathworks.com . Accessed 24 July 2024

[24] Terashima Y, Yurube T, Hirata H, Sugiyama D, Sumi M, Hyogo Organization of Spinal D (2017) Predictive risk factors of cervical spine instabilities in rheumatoid arthritis: a prospective multicenter over 10-year cohort study. Spine (Phila Pa 1976) 42(8):556–564. 10.1097/BRS.0000000000001853 27525538 10.1097/BRS.0000000000001853

[25] Ahn JK, Hwang JW, Oh JM et al (2011) Risk factors for development and progression of atlantoaxial subluxation in Korean patients with rheumatoid arthritis. Rheumatol Int 31(10):1363–1368. 10.1007/s00296-010-1437-y 20422194 10.1007/s00296-010-1437-y

[26] Pisitkun P, Pattarowas C, Siriwongpairat P, Totemchokchyakarn K, Nantiruj K, Janwityanujit S (2004) Reappraisal of cervical spine subluxation in Thai patients with rheumatoid arthritis. Clin Rheumatol 23(1):14–18. 10.1007/s10067-003-0782-6 14749975 10.1007/s10067-003-0782-6

